# Whole community invasions and the integration of novel ecosystems

**DOI:** 10.1371/journal.pcbi.1010151

**Published:** 2022-06-07

**Authors:** Colin Campbell, Laura Russo, Réka Albert, Angus Buckling, Katriona Shea

**Affiliations:** 1 Department of Biochemistry, Chemistry, and Physics, University of Mount Union, Alliance, Ohio, United States of America; 2 Department of Ecology & Evolutionary Biology, University of Tennessee, Knoxville, Knoxville, Tennessee, United States of America; 3 Department of Physics, Pennsylvania State University, University Park, Pennsylvania, United States of America; 4 Department of Biology, Pennsylvania State University, University Park, Pennsylvania, United States of America; 5 Department of Biosciences, University of Exeter, Penryn Campus, Penryn, Cornwall, United Kingdom; University of Chicago, UNITED STATES

## Abstract

The impact of invasion by a single non-native species on the function and structure of ecological communities can be significant, and the effects can become more drastic–and harder to predict–when multiple species invade as a group. Here we modify a dynamic Boolean model of plant-pollinator community assembly to consider the invasion of native communities by multiple invasive species that are selected either randomly or such that the invaders constitute a stable community. We show that, compared to random invasion, whole community invasion leads to final stable communities (where the initial process of species turnover has given way to a static or near-static set of species in the community) including both native and non-native species that are larger, more likely to retain native species, and which experience smaller changes to the topological measures of nestedness and connectance. We consider the relationship between the prevalence of mutualistic interactions among native and invasive species in the final stable communities and demonstrate that mutualistic interactions may act as a buffer against significant disruptions to the native community.

## Author summary

The interactions between species in an ecological community can be significantly perturbed by the introduction of new (i.e., invasive) species. The impact of species introductions can be very challenging to predict, making it difficult to manage or prevent undesirable consequences (e.g., decreased crop pollination). In this report we simulate community invasion by sets of plant and pollinator species that either *do* or *do not* represent a stable community to generate predictions about the impacts on interactions in a native community. We find important differences in outcomes including, for instance, that merging two *stable* communities is less detrimental to the native community than the introduction of an equal number of *random* invasive species.

## Introduction

The invasion of non-native species can drive significant changes in ecosystem biodiversity and functioning, for instance by influencing species richness [1,2], composition [3], native plant growth rates [4], altering the structure of local food webs [5–7], introducing disease [8], driving the regional loss of some species [9], altering patterns of species-species interactions [10], or influencing the likelihood of fire [11]. Understanding the impacts of species invasions is of particular import in the context of community reconstruction projects [12], especially in light of escalating rates of extinction and colonization [13], and increasing recognition that perturbations to a community can cascade due to a positive feedback loop where “change begets change” [14].

It is challenging to achieve this understanding, though, because ecosystem responses to a given invasion event are highly dependent on the characteristics of the invading species along with the evolutionary history and composition of the target community [15]. As such, community response to invasion–which can occur rapidly–can be challenging to predict [16]. Indeed, even absent invasions, communities are inherently dynamic; species interactions can change over time and the position of a species in the broader network of community interactions can shift [17–19]. Driven by these challenges, the study of invasion science has received significant attention (e.g., [9,20–23]). Understanding the assembly history of a community (including the original status of current resident species as native or invasive) is increasingly recognized as important for understanding its diversity, composition, function and response to invasion (e.g., [24–26]).

While invasion studies often consider a single invasive species, groups of species can invade a native community simultaneously [27–34]. A particularly interesting example of multiple species invasion is *stable community invasion*, where the invading species themselves compose a stable (or *whole*) community separate from the native community. Examples include anaerobic digestor microbial communities, gut microbiomes (e.g. fecal transplants), aquatic invasions (e.g. from seawater ballast), and soil transplant [35–39].

Here we focus on communities of plants and pollinators, constituent species of which can move via natural dispersal or range expansion [40,41], or through human intervention [42,43]. Invasive species can dramatically impact native communities [44,45], and the extent of invasion can vary significantly [46,47]. Invasion in this context is especially relevant with regard to disturbance [48] and the formation of novel ecosystems [47]; the common origin of non-native species can influence their behavior in a new community [49].

For example, urban landscapes tend to comprise a large proportion of both non-native plants and non-native pollinators, which may preferentially interact with one another [50]. These urban plant-pollinator networks integrate and/or invade into native networks of plants and pollinators where urban land use is expanding, and over longer time scales across large areas of the landscape, creating novel ecosystems [51,52]. Another example is where pollinators have been introduced deliberately or accidentally that provide crop pollination services. The crop species themselves are typically not native to the area where they are grown and are accompanied by a set of field edge typical weedy plant species that tolerate agricultural disturbance well [53]. Alongside these, the introduced pollinators tend to prefer the non-native plant species [54] and can supplant or integrate with the native pollination networks [46]. In these cases, it is critically important to understand the relative impacts of introducing species as a collective, functional community or as separate invasive species without established prior interactions or other community structure [55].

When multiple species co-invade a community, the effect can be (a) a competitive (less than additive), (b) neutral (additive), or (c) facilitative (greater than additive) impact due to synergistic interactions [29,56–58]. A meta-analysis has shown that the combined impact of multiple animal invaders on species’ performance is typically less than additive, though exceptions are common and the details complex [31]. Thus, it is clear that understanding the total impact of multiple invasive species is critical for effectively managing invaded communities [29,31,59]. For instance, removing an invasive species may facilitate subsequent invasion by a different species [60].

Many predictive models have been developed to characterize the invasion process and identify optimal detection/control strategies, including spatially explicit spreading models [61] and models based on niche theory and/or stochastic dynamics [62–66]. Some models explicitly consider multi-species invasions (e.g., [67–69]); while some parallels exist between models of community assembly (e.g. [62,70–73]) and multi-species invasion, the situations are distinct in part because a non-native invader may have characteristics drastically different than the native population. Recent work has also considered the formation of a community from two parent communities in the context of single trophic level microbial communities; in this context the process is sometimes referred to as *coalescence* [74–76].

In the context of multi-species invasion, there is a particular need to better understand the implications of whole (i.e., stable) community invasion for resident species persistence and community structure/stability. Networks are a natural and attractive framework for modeling ecological communities because data on species-species interactions (i.e., network structure) can be determined empirically and network models can relate community structure to emergent dynamic behavior (e.g., the “ripple effect” as the introduction of an invasive species affects native species with which it does not directly interact).

**Fig 1:**
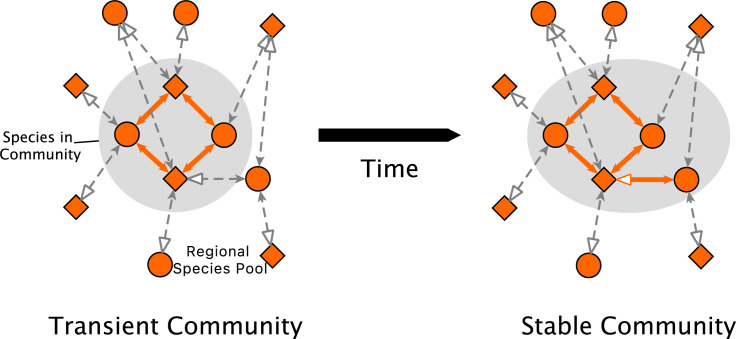
The community assembly model of Campbell et al. [72]. Interactions between plants (diamonds) and pollinators (circles) are shown with arrows; beneficial interactions are shown with filled arrowheads and detrimental interactions are shown with unfilled arrowheads. Species present in the community are shown inside the gray shaded region; species in the regional species pool (i.e., species that may colonize if conditions become favorable) are positioned outside the shaded region. Interactions involving species in the regional species pool do not affect species in the community of interest and are shown with dashed gray connections; interactions between species in the regional species pool are omitted for visual clarity. (Left) An example of a transient (i.e., unstable) community consisting of two plant species and two pollinator species present in the community. While the four species are self-sufficient, one additional pollinator species from the regional species pool is poised to colonize the community by virtue of its beneficial interaction with the bottom plant species. (Right) According to the dynamic update process described in Eq [1], the pollinator species is able to colonize the community on the subsequent time step. This community is considered stable because no other colonization or extinction events will occur from this configuration.

In this work, we consider whole community invasion in the context of a network-based model originally developed in the context of plant-pollinator community assembly [72]; the model is sketched in Fig 1 and summarized in the Methods. The model generates an ensemble of plant species and pollinator species, called the *regional species pool*, that have interaction patterns (e.g., the relative abundance of mutualistic interactions) that follow empirical distributions. Random sets of species are drawn from the regional species pool and placed in a community of interest; the interactions between these species determines if they persist (e.g., if a plant species is pollinated) or become locally extinct. Similarly, species in the regional species pool but absent from the community of interest colonize if the situation is favorable (e.g., if a pollinator identifies sufficient food). This dynamic colonization & extinction process continues until a so-called *stable community* is formed, where either (a) no further changes to community composition occur (in the language of dynamical systems, such a community composition is referred to as a *steady state*) or (b) any changes occur cyclically and predictably (a *limit cycle*). The term *stability* has multiple meanings in the ecological literature; we emphasize that we here use the term to refer to the static or near-static composition of the community rather than as a measure of the community’s response to a perturbation or the numerical abundance of present species. For a study of stability in this sense in the present model, see [77]; for a recent review of ecological stability see [78].

**Fig 2:**
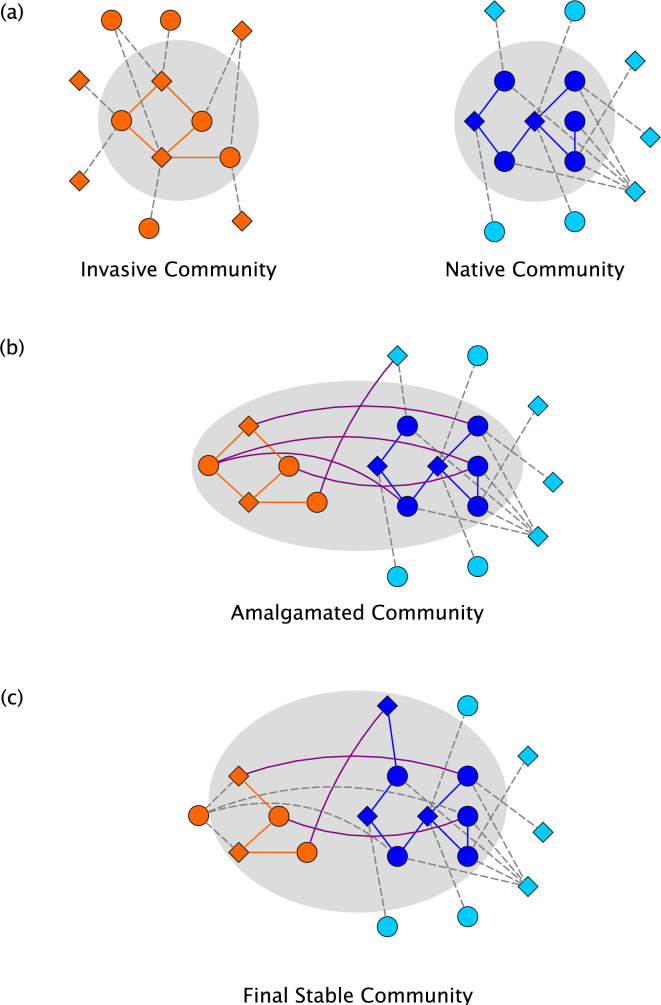
The invasion process and types of communities considered in this report. For simplicity in this schematic example, the arrowheads describing the nature of the interactions (Fig 1) are omitted. (a) Both the invasive community and the native community are assembled via the process described in Fig 1. (b) The amalgamated community includes the species present in the invasive community, the species present in the native community, and the native community’s regional species pool. Interactions between species in the invasive and native communities, which were irrelevant prior to the invasion, are here shown with curved edges. (c) After the invasion represented in panel (b), the community assembly process continues according to Eq [1]. Here we indicate the local extinction of one invasive species (on the far left of the panel) and the colonization of one species from the regional species pool (at the top of the panel).

We extend this model by merging independent stable communities, one considered *native* and the other *invasive*, and tracking the ensuing dynamics as the *amalgamated* community reaches a *final stable* community (Fig 2). We compare this whole community invasion scenario to a multiple species invasion scenario where the invaders do not themselves represent a stable community. We show striking differences in the final stable communities under these two contrasting invasion scenarios; for instance, under whole community invasion, species present in the amalgamated community are more likely to exist in the final stable community compared to random species invasion. These findings characterize–and highlight the importance of understanding–the relationship between the assembly history of invasive species (here, specifically plants and pollinators) and their impact on native communities.

## Results

### Community size

**Fig 3:**
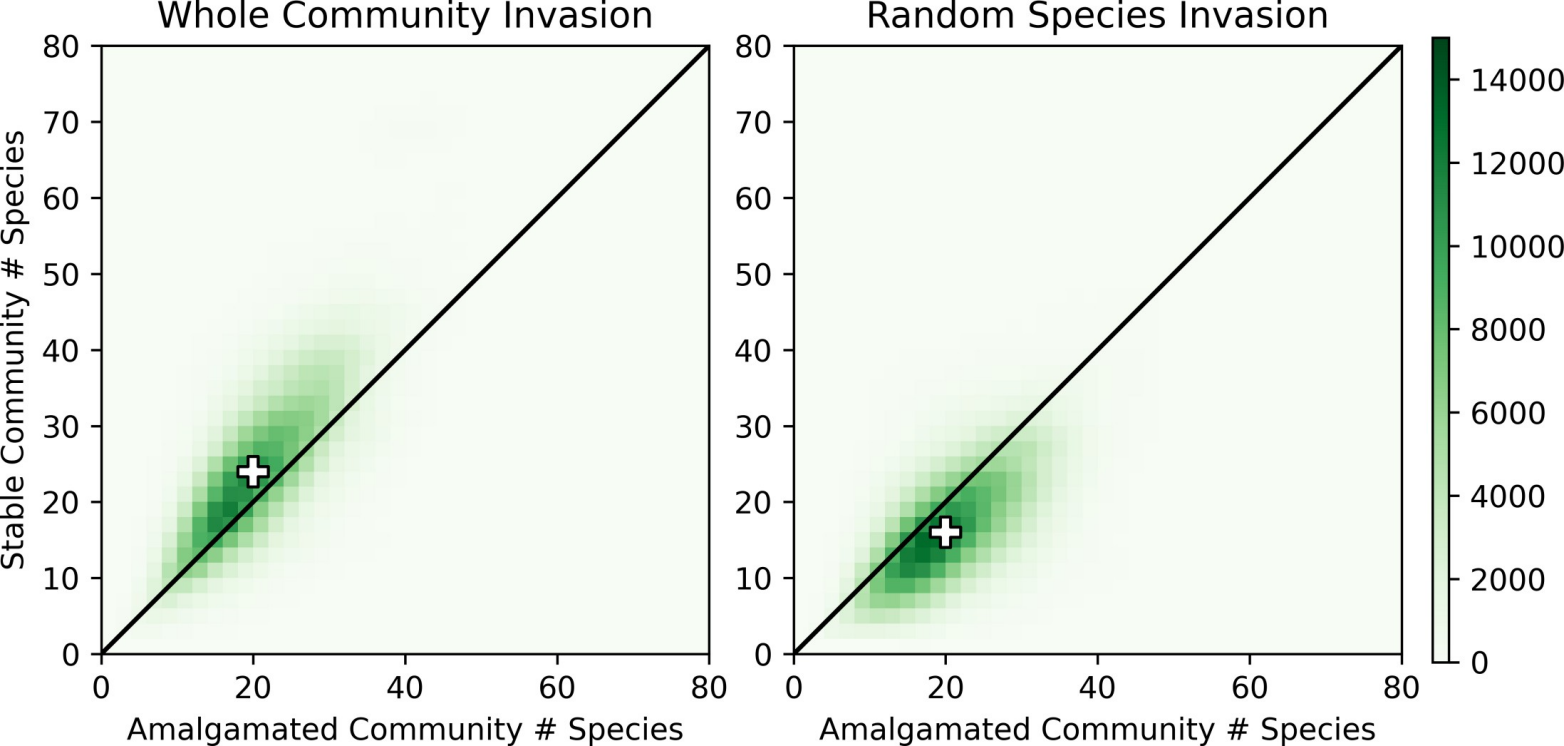
The effect of multi-species invasion on species richness. The horizontal axes indicate the total number of native and invasive species at the time of invasion (i.e., in the amalgamated community); the vertical axes indicate the total number of species in the resulting attractor (stable state or limit cycle). Coloring indicates the number of simulations with the corresponding species counts. The black line indicates a 1:1 ratio for visual reference and the “+” symbol indicates the median. When the invaders form a stable community (left), the species richness generally increases. In contrast, if the invaders do not form a stable community (right), the species richness generally decreases.

We find that invasion by a stable community generally *increases* species richness compared to the total number of native and invasive species in the amalgamated community; i.e., invasion by a stable community of nonnative species facilitates colonization by species from the regional species pool. In contrast, invasion by a random group of species generally *decreases* species richness (mean change of 21% and -15%, respectively; Fig 3). We considered the role of the sizes of the communities by determining the Spearman correlation coefficient between the number of species in the final stable community and (a) the number of invading species, (b) the number of species in the native community, and (c) the total number of invading and native species in the amalgamated community.

In the case of whole community invasion, we found that the number of species in the final stabilized community was most strongly correlated with the total number of species in the amalgamated community (0.72 vs. 0.52 for invasive species only and 0.41 for native species only). This suggests that when one stable community invades another, larger groups of species can lead to larger stable final communities regardless of the relative sizes of the two source communities.

In contrast, when considering random invasions, the number of species in the final stable community has similarly high correlations with both the number of species in the amalgamated community (0.60) and the number of native species (0.62), with only a weak correlation with the number of invasive species (0.13). The drop in this last correlation compared to whole community invasion indicates the disproportionate impact of the organization of the invasive species in determining the species richness of final stabilized communities.

A recent study [79] found that stable communities in this model are anchored by network motifs–small sets of species fixed in the “present in the community” or “absent from the community” state–that tend to have mutualistic (mutually beneficial) interactions. Accordingly, we hypothesized that a greater abundance of mutually beneficial interactions in the amalgamated community may support a larger final stable community. Interestingly, we found that the fraction of species interactions in the amalgamated community that were mutually beneficial was negatively correlated with the size of the final community (-0.24 for whole community invasion and -0.07 for random species invasion). Thus, while whole community invasion can drive a more significant increase in community size than random species invasion, mutually beneficial interactions in the source communities may mitigate this growth.

### Relative abundance of native and invasive species

**Fig 4:**
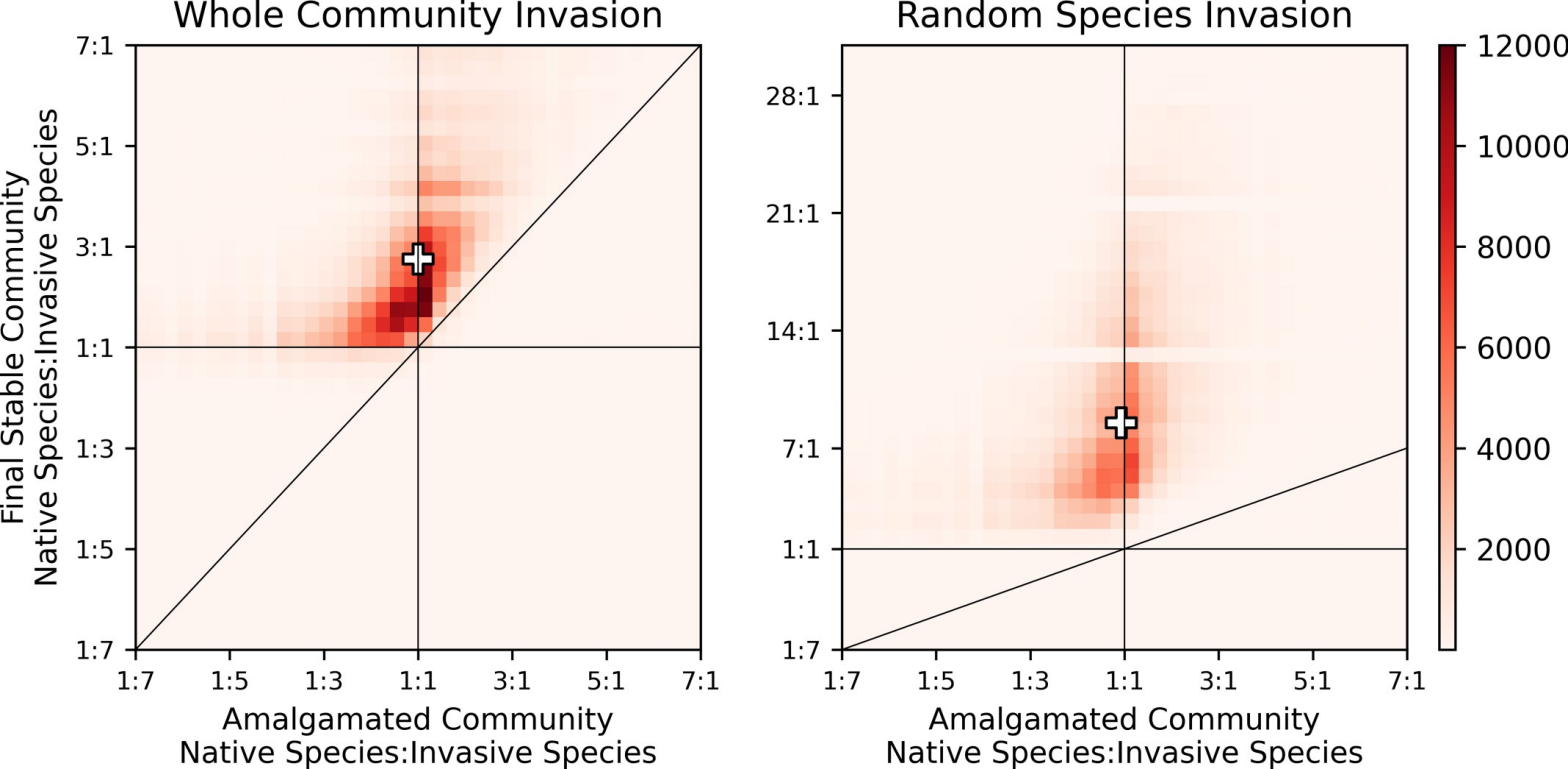
The symmetry between native and invasive species from the amalgamated community (horizontal axes) to the final stable community (vertical axes). Note the different ranges on the vertical axes to capture the long tail in the case of random species invasion. Thin black lines are included to guide the eye; the angled line indicates constant symmetry from the amalgamated community to the final stable community (i.e., it has a slope of 1). The “+” symbol indicates the median. For both whole community and random species invasion, the tendency is for final communities to become native dominant, though the effect is much less pronounced in the case of whole community invasion.

We also considered the symmetry (e.g., [80]) between native and invasive species in the amalgamated and final stable communities (Fig 4). Because all possible community combinations are considered when simulating invasions, the median ratio of native to invasive species in the amalgamated communities (shown on the horizontal axes on Fig 4) is 1:1. For nearly all invasions, however, the final community has more native species than invasive species. Notably, the effect can be more dramatic for random species invasion compared to whole community invasion. These results indicate that invasion by species that represent stable communities is more likely to lead to colonization by the invasive species (insofar as the invasion can lead to final stable communities with a ratio of native to invasive species less skewed toward native species) compared to invasion by randomly selected species.

### Species persistence

**Fig 5:**
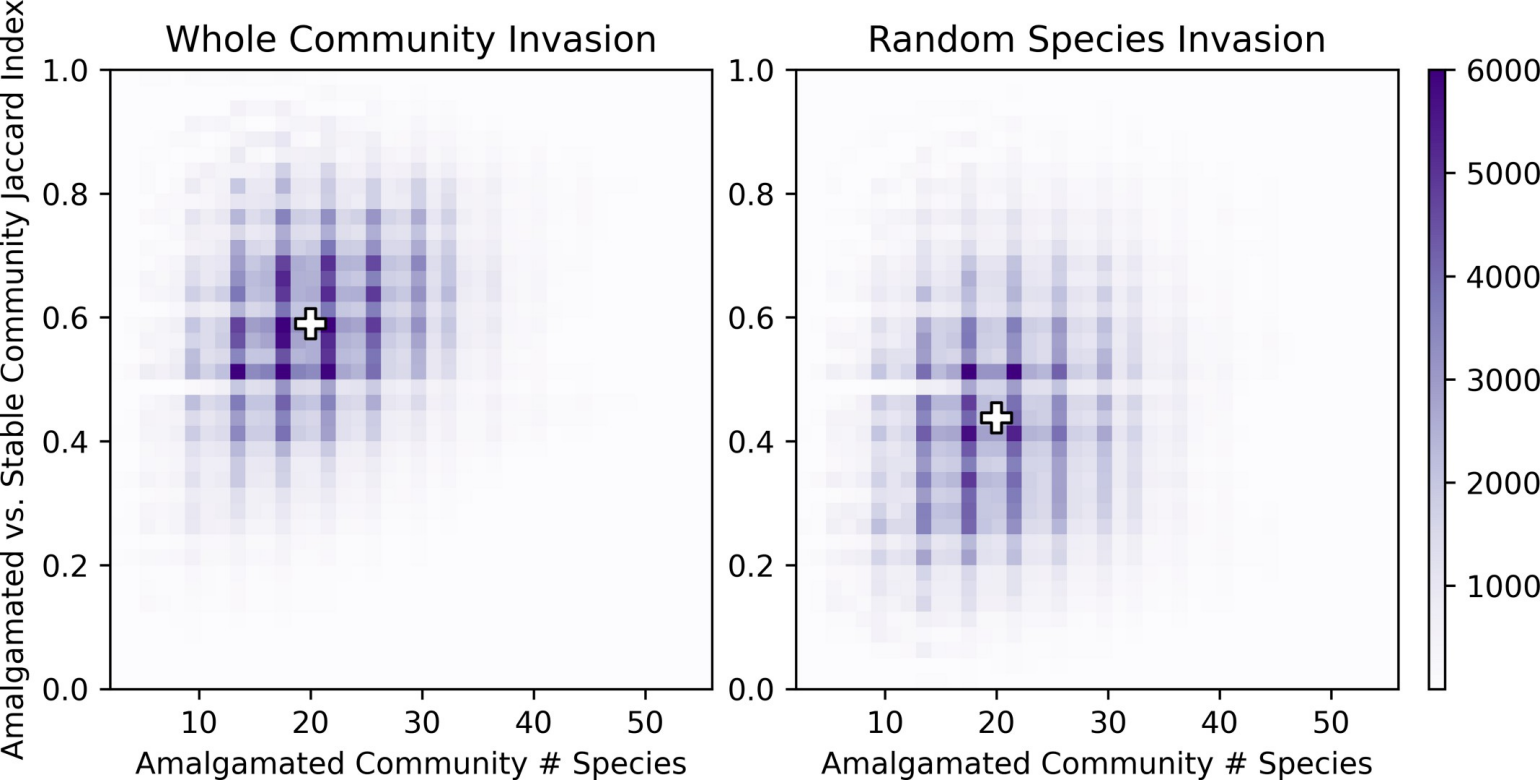
The Jaccard index vs. the total number of native and invasive species at the time of invasion. The “+” symbols indicate the medians. The Jaccard index tends to be greater when the invaders form a stable community (left) compared to when the invaders do not form a stable community (right), indicating that whole community invasions result in less species turnover.

In addition to the *size* and *symmetry* of the communities, we also considered the *similarity* of the amalgamated community with the final stabilized community using the Jaccard index: the ratio of the number of species that exist in both the amalgamated and final stable communities (i.e., the size of the intersection of the two sets) to the total number of species that exist in one or both of the communities (i.e., the size of the union of the two sets). The Jaccard index varies between 0 for communities with no shared species to 1 for identical communities. Whole community invasion, despite showing a greater change in species richness than random species invasion, preserves the composition of the amalgamated community to a greater extent than invasion by random species (mean Jaccard index of 0.58 for whole community invasion vs. 0.45 for random species invasion, Fig 5). We find that native species are typically able to persist in the final stable community, regardless of invasion type (average of 93% for whole community invasion and 91% for random species invasion), indicating that the higher Jaccard index for whole community invasion is due to the greater likelihood of survival by the invasive species compared to random invasion. (Note that this finding is in agreement with the more balanced native to invasive species ratio for whole community invasion observed in Fig 4.)

The Jaccard index is also related to the abundance of mutually beneficial interactions in the amalgamated communities; more mutually beneficial interactions are weakly correlated with the Jaccard index (Spearman correlation coefficient of 0.04 for whole community invasion and 0.24 for random species invasion) but tend to be weakly anticorrelated with the number of native and invasive species in the final stable communities (Spearman correlation coefficient of -0.21 and 0.06 for native species in whole community and random species invasion, respectively; for invasive species the correlations are -0.24 for whole community invasion and -0.23 for random community invasion).

### Network properties

**Fig 6:**
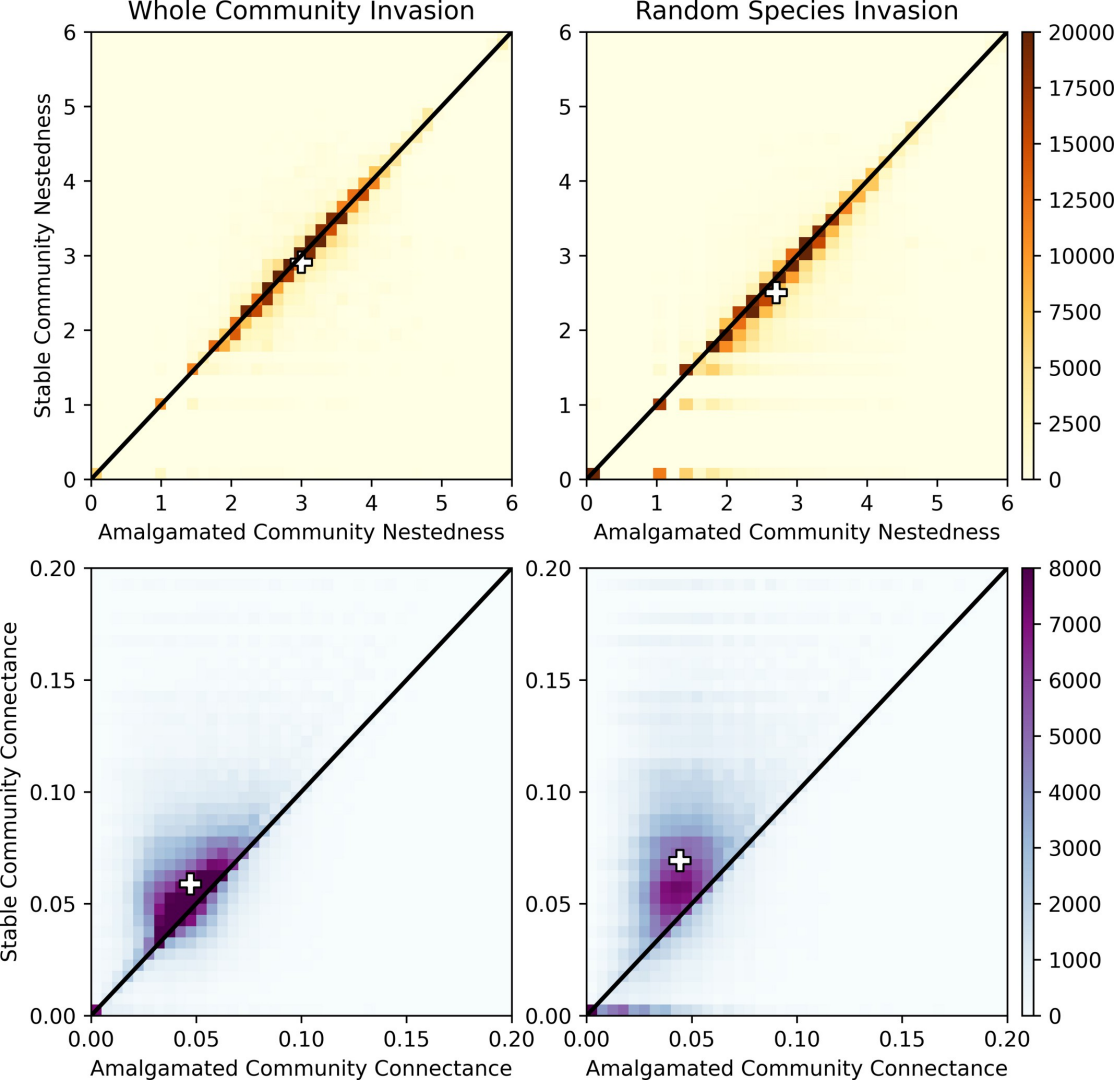
The nestedness (top) and connectance (bottom) of amalgamated vs. final stable communities; the figure shows invasion by both stable communities (left) and a random selection of species (right). Black lines show a 1:1 ratio for visual reference and the “+” symbols indicate the medians. For both invasion types the nestedness decreases slightly as a result of the invasion and the connectance increases; for both measures the shift is greater in the case of random species invasion.

We also considered the role of spectral nestedness [81] and connectance in merged and stabilized communities (Fig 6; see Methods for definitions). The differences in the merged vs. stabilized distributions for both measures in both invasion scenarios were statistically significant according to two-sample Kolmogorov-Smirnov tests (*p*<10^−10^). We found that nestedness decreased slightly for both types of invasions, though more so for the random invaders (2.70 when merged vs. 2.47 after stabilization) than whole community invasions (2.97 vs. 2.89). In contrast, connectance increased for both types of invasion; again, the change was greater for random invaders (0.048 vs. 0.083) than whole community invasions (0.050 vs. 0.068).

**Fig 7:**
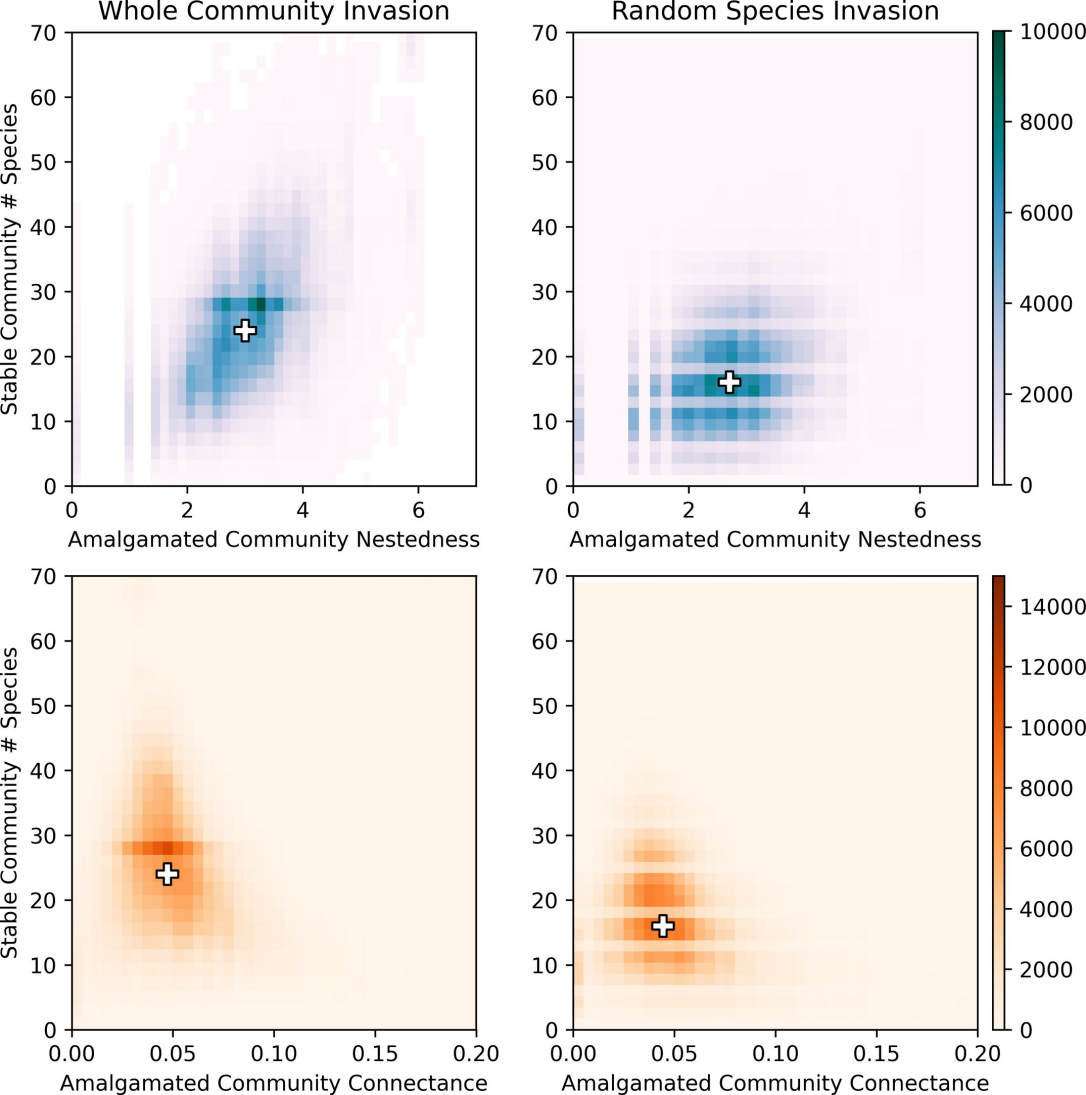
The nestedness (top) and connectance (bottom) of amalgamated communities vs. final stable community size; the figure shows invasion by both stable communities (left) and a random selection of species (right). The “+” symbols indicate the medians. Nestedness is positively correlated with community size (more so for whole community invasion than for random species invasion), while connectance is negatively correlated with community size.

Nestedness was positively correlated with the size of the stable community (Spearman correlation of 0.53 for whole community invasion and 0.24 for random species invasion), while connectance was negatively (albeit weakly) correlated with the size of the stable community (Spearman correlation of -0.24 and -0.19 for whole community and random species invasion, respectively); see Fig 7. These findings differ from single species invasions, where an increase in species richness generally corresponded to increases in nestedness and decreases in connectance [82], suggesting that the impact of invasion by multiple species is not simply an additive effect from multiple individual species.

### Summary

Taken together, our results indicate that whole community invasion facilitates the persistence of invaded and native species *and* colonization of species from the native regional species pool to a greater extent than invasion by equal numbers of random species. We also found that key network metrics are more stable when considering whole community invasion and that mutually beneficial interactions may act as a (weak) buffer against disruption to community composition at the cost of decreased community size.

## Discussion

The increasing rate of biological invasions [83,84] highlights the need for ecologists to better understand the effects of invasion by multiple species on ecosystem function and community properties including, for instance, pollination rates, changes to the overall structure and composition of the community, and the resistance of the community to future invasion. In this context, a tempting null hypothesis is to suppose that multiple previously interacting invader species have the same effect as multiple individual invaders (that did not previously interact, but instead are brought together *de novo* to a new community). However, this implies that a prior history of interactions has no effect on future community structure. There is increasing evidence that this is not the case, and that history and legacy do matter for invasion and diversity [26,85,86]; our research addresses the role of mutualistic interactions in the dynamics of communities.

Here we have considered the role of invader history by leveraging a model originally developed in the context of plant-pollinator community assembly [72]. By identifying stable communities (in the sense that, absent perturbation, the species in the community coexist with either no turnover or minimal and cyclic turnover) and then simulating situations where they are invaded by either (a) other stable communities or (b) randomized groups of species, we investigated the role of the history of the invasive species. Our results indicate that the history of the invasive species does play an important role in the post-invasion dynamics; when the host community is invaded by species that themselves constitute a stable community, the collection of native and invasive species are more likely to persist in the ensuing stabilized community compared to the case where the invaders have no interaction history (i.e., random species assemblages invade less well than whole community assemblages). Invasion by stable communities also generally leads to an increase in species richness (i.e., outside species from the native regional pool are more likely to colonize the merged community), in contrast to the overall decrease in species richness typical of invasion by collections of random species. Furthermore, the nature of species-species interactions in the native and invasive communities appears to play a mechanistic role in shaping community response, as mutually beneficial plant-pollinator interactions may act to preserve community composition and mitigate community growth.

In addition to species richness, plant-pollinator networks are commonly characterized by their connectance and nestedness. These topological measures are entangled with one another and are frequently studied in the context of community stability (for example, in response to the sudden loss of one or more species). Nestedness is promoted by niche exploitation–modulated by adaptive foraging–but maximally nested communities are over-reliant on a subset of species and are vulnerable to collapse; thus, stable communities are typically nested but not *perfectly* nested [77,81,87]. A community with an exceedingly low connectance is unstable, as species without interacting partners go extinct. In contrast, a community with a very high connectance cannot be nested, as larger connectance values correspond to more species acting as generalists; thus, stable communities tend to have low but nonzero connectance values. However, these measures do not *determine* community stability and small variations (for example, in response to invasions) should not, *ipso facto*, be interpreted as an indication of altered community stability. Nonetheless, connectance and nestedness are useful for characterizing the extent to which a community is reshaped in response to invasion or other perturbation.

Our results indicate that invasion by whole communities tends to drive a decrease in nestedness that is less severe than the decrease in nestedness driven by random species invasion. The fact that greater changes to both nestedness and connectance are observed for random species invasions suggests that the corresponding amalgamated communities are further from equilibration than their whole community invasion counterparts.

Each of the topological measures (species richness, nestedness, and connectance) considered here supports the hypothesis that plant-pollinator communities invaded by whole communities are more resilient to subsequent invasion than communities invaded by random groups of species. In general, a larger native community leads to a final stable community that is both larger and more similar to the native community, suggesting that it may be more likely to retain species in the event of additional multi-species invasions.

An obvious area for future work is to test these hypotheses in an empirical context [88,89]. In the context of plant-pollinator communities, this could be accomplished by exploring how networks change in response to urbanization, which tends to introduce and promote sets of interacting species of plants and pollinators, relative to nearby natural areas where only the highly mobile pollinators species have invaded. Separately, because the model employed in this report considers plant-pollinator interactions, our findings should at most be considered suggestive rather than predictive when considering whole community invasion in other ecological contexts. A particularly exciting area for future work is whole community microbial invasions, which have direct relevance to clinical and agricultural health, and biotechnology (e.g., [32,80]). Complementary studies of invasion in different ecological contexts promise to provide insight into universal versus context-dependent behavior.

## Methods

### The community assembly model

We here provide an overview of the community assembly model of Campbell et al. [72]. The model begins by forming a *regional species pool* with a prescribed number of plant species and pollinator species. We emphasize that the regional species pool is not itself a stable community. Rather, it represents the collection of species (and their interactions) that can be present in a community of interest; a stable community includes a subset of the species from the regional species pool. Indeed, during community assembly, species colonize the community of interest from the regional species pool, persist in the community, or go extinct in a dynamic process that depends on interactions between currently present species that can be beneficial or detrimental, for example due to pollination or nectar robbing (Fig 1).

To characterize species-species interactions, each species is assigned (a) a characteristic length (a proboscis length for pollinators and a nectar depth for plants) and (b) the collection of species with which it interacts; both values are probabilistically assigned according to empirical distributions [90,91]. Each plant-pollinator interaction is then categorized as *mutually beneficial* if the characteristic lengths are similar (the pollinator feeds while the plant is pollinated; here we follow previous work in considering interactions with ≤10% difference as mutually beneficial). Otherwise, the interaction is beneficial to the species with the larger length and detrimental to the other species: either the plant is pollinated but the pollinator is unable to draw sustenance or the pollinator feeds without pollinating the plant.

Once constructed, the regional species pool can be represented by a bipartite *interaction network* where each species is represented by a node and species-species interactions are represented by signed bidirectional edges (Fig 1). The interaction network serves as the substrate upon which dynamical processes (e.g., community assembly and invasion) occur. Dynamics are considered in a Boolean framework where species are either abundant in the local community (logically ON) or not (logically OFF). As such, in a network with *N* species there are 2^*N*^ possible combinations of species. We consider each combination to be a *network state* and the process by which the community dynamically moves between these states (i.e., as some species invade from the regional species pool and others go locally extinct), eventually reaching a stable community configuration, can be summarized in the *state transition network* (Fig 1).

The transition from one network state to the next is determined by the net effect of each species’ currently present interacting partners: if overall beneficial (e.g., there exists an abundant food supply for a pollinator species), then a species successfully enters (if previously absent) or remains in the community (if previously present) and is denoted as ON in the next state. Otherwise, the species is denoted as OFF (the species fails to colonize or, if present at time step *t*, its population drops to a negligible level at time step *t*+1). Mathematically, the state of a node *i* at time step *t*+1 is determined by

$$S_{i}(t+1)=\{\begin{array}{ll} 1, & \sum_{j}S_{j}(t)E_{ji}\geq 1\\ 0, & \mathrm{otherwise} \end{array}$$ [1]

Where the sum is taken over all species that interact with node *i*, *S*_*j*_(*t*) denotes the state of interacting species *j* at time step *t* (1 if the species is ON and 0 if the species is OFF), and *E*_*ji*_ denotes the weight of the interaction between node *j* and *i*. In short, for a species to be present at time step *t*+1, the net effect of the present species with which species *i* interacts at time *t* must be positive. Here we follow previous work [69,72,77,79,82,92] and set positive interactions to have a weight of 4 and negative interactions to have a weight of -1, which means that a beneficial interaction enables a species to tolerate several negative interactions. The reason for setting positive weights to a greater value than negative weights is to account for the fact that, in nature, the positive effect of successful pollination (for a plant) or feeding (for a pollinator) outweighs the loss of nectar (for a plant in the context of nectar robbing) or time (by a pollinator unsuccessfully attempting to feed).

This observation does not, however, suggest appropriate numerical values for the edge weights; indeed, it is challenging to *empirically* quantify the relative costs and benefits of species interactions. Previous computational investigations with this model have considered fixed negative edge weights of -1 and positive edge weights at or above +1, and demonstrated that as long as the positive edge weight has a greater magnitude than a negative edge weight, (a) the number of stable communities for a given regional species pool does not vary significantly and (b) the number of species present in a stable community tends to increase as the positive edge weight increases [72]. A study of community response to the introduction of an invasive species considered both typical and atypical interactions (e.g., invasive species whose positive interactions were weighted as high as +8 compared to a standard positive weight of +4). The invader characteristic was found to have minimal impact on the community’s topology, as measured by connectance and nestedness, during the post-invasion re-equilibration process [82]. In sum, then, previous work indicates that the specific choice of edge weights has little impact in the ensuing community dynamics aside from ensuring the initial stable communities are of sufficient size to be of ecological interest. Here, we choose positive weights of +4 and negative weights of -1 to provide consistency with the previous investigations regarding this model. In the S1 Appendix we report the results of complementary analysis with positive weights of +3 and +5 and confirm that the general model behavior reported in the main text is robust to these variations.

The update process is performed *synchronously*, meaning the state of a node at time *t*+1 is determined by the states, at time *t*, of the nodes with which it interacts. To identify the stable communities, we randomly sample the 2^*N*^ states and advance the dynamics until a so-called *attractor* is reached. An attractor is either a *stable state*, where the state of every species remains fixed across subsequent updates, or a *limit cycle*, where the state of the network advances through a repeating subset of states. Sampling a sufficient number of states captures most or all of the attractors of the interaction network [72], though we note that a recent study has identified computationally efficient methods for identifying the complete repertoire of attractors [79]. The network properties of the stable communities are similar to empirical communities, and the model has previously provided insight to, for instance, invasion by a single species [82].

### Whole community invasion and multiple species invasion

For this study we generated 1000 interaction networks, as described above, each with 50 plant species and 150 pollinator species [93]. To identify isolated stable communities, we then split each interaction network into two separate regional species pools, each with 25 plant species and 75 pollinator species, such that inter-community interactions are inactive (i.e., irrelevant to community dynamics) until an invasion event occurs. Stable communities in these isolated interaction networks were identified by randomly sampling 100 initial states and advancing the dynamics until an attractor was found. This procedure allowed us to generate independent communities, mimicking geographically isolated regions, while still forming species-species interactions between the communities that become relevant during invasions.

We then considered whole community invasion by selecting one stable community from each of the interaction network halves. One community is designated the *invader community* and the other the *native community*. The communities are then merged, i.e., we consider the network state where a species is ON if it is ON in either parent community. In cases of limit cycle parent communities, one constituent state was randomly chosen to serve as the parent for the purposes of state combination. The dynamics were advanced from the combined state until a new attractor (the *final stable* community) was identified. During this process, species in the invader community’s regional species pool were unable to colonize the community of interest to reflect the fact that the invader community has been moved from its native locale and its regional species pool cannot interface with the native community or its regional species pool. We exhaustively considered all possible whole community invasions across the 1000 interaction networks (i.e., all pairs of stable communities were merged in each interaction network) for over 800,000 simulated whole community invasions.

We also considered invasions by multiple species in cases where the invaders did not constitute a stable community. Each whole community invasion described above was partnered with a separate invasion where the species in the invader community were randomized prior to the invasion. Again, species in the random invading species’ regional species pool were unable to colonize the community of interest to reflect the fact that the invading species have been moved from their native locale and thus the regional species pool from which the invasive species are drawn cannot interface with the native community. This allowed us to control for the number of invasive species when comparing the effect of stable vs. non-stable groups of invasive species.

### Network metrics

In addition to the number and identities of species in the amalgamated and final stable communities, we also considered the interrelated topological measures of connectance and nestedness. Connectance is a low-level metric that measures the fraction of possible species-species interactions that are observed; for a community with *M* plants and *N* pollinators, the total number of possible interactions is *MN*. Nestedness is a higher-level metric that, broadly speaking, measures the extent to which the community consists of generalists (species with many interacting partners) and specialists (species with few interacting partners) that tend to interact with generalists. In this report we use the spectral nestedness measure of Staniczenko et al. [81], which calculates nestedness from the eigenvalue spectrum of the adjacency matrix that describes species-species interactions in a community.

## Supporting information

S1 Appendix An analysis of the model’s sensitivity to the weight of positive plant-pollinator interactions. While Figs 3–7 in the main text consider a positive edge weight of 4, Figs A-G in S1 Appendix show parallel results for positive edge weights of 3, 4 (for ease of comparison to the main text), and 5.
(DOCX)
