# Supplementary material for: Whole community invasions and the integration of novel ecosystems
Source: PLoS Comput Biol. 2022 Jun 7;18(6):e1010151. doi: 10.1371/journal.pcbi.1010151 (PMC9173635; doi:10.1371/journal.pcbi.1010151)
Supplement: S1 Appendix — While Figs 3–7 in the main text consider a positive edge weight of 4, Figs A-G in S1 Appendix show parallel results for positive edge weights of 3, 4 (for ease of comparison to the main text), and 5. (DOCX) [file pcbi.1010151.s001.docx]

**Whole Community Invasions and the Integration of Novel Ecosystems**

Supplementary Information

Colin Campbell, Laura Russo, Réka Albert, Angus Buckling, and Katriona Shea

In the main text we consider the behavior of the model where beneficial interactions between plants and pollinators carry weight +4 and detrimental interactions carry weight -1 (see Materials and Methods for justification). Here, we perform sensitivity analysis on these findings by repeating all simulations with beneficial interaction weights of +3 and +5.

The figures shown below correspond to Figures 3-7 in the main text; we have matched the color scheme from the corresponding main text figure and included the *+*4 results (from the main text) for ease of comparison. In labeling the figure panels we denote the positive edge weight as *p*. In summary, our results are consistent across all three edge weights considered in the sensitivity analysis.

**
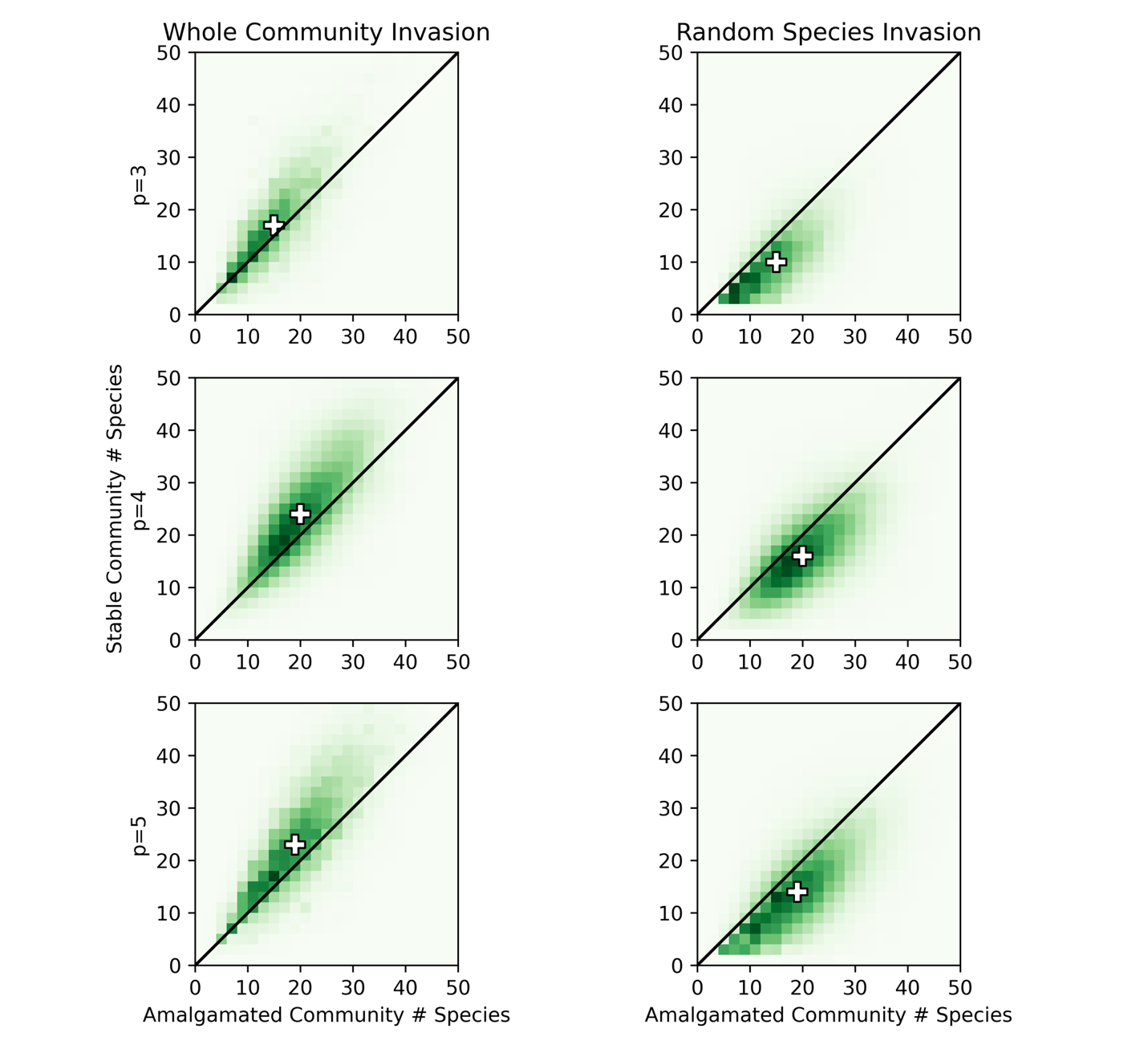
**

**Figure A.** Amalgamated community species richness vs. stable community species richness. The middle row panels show the same data as Figure 3 in the main text. The “+” symbols indicate the medians. The trend where whole community invasion (random species invasion) drives an increase (decrease) in species richness is consistent for all three considered edge weights.

**
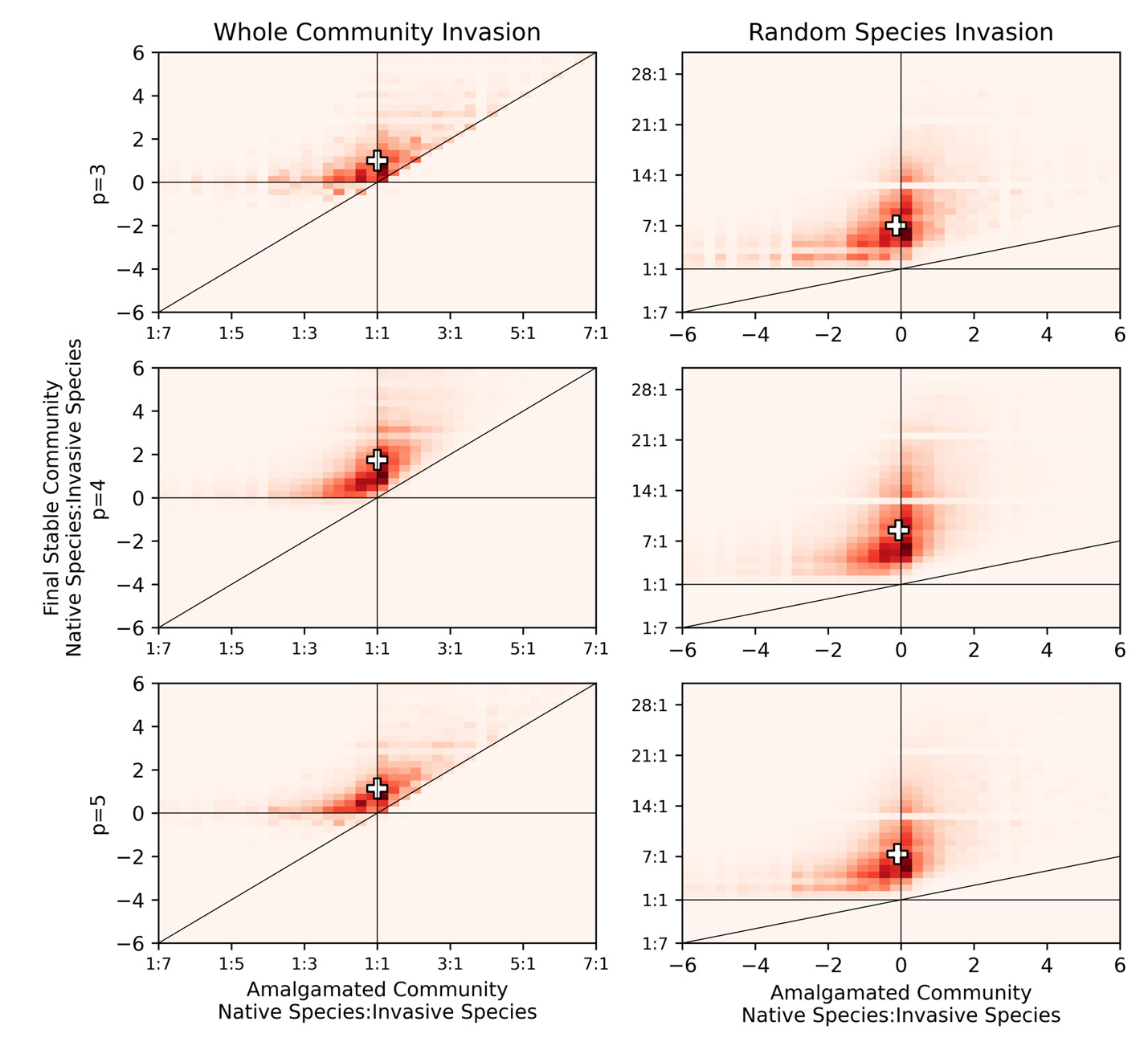
**

**Figure B.** The symmetry between native and invasive species from the amalgamated community to the final stable community. The middle row panels show the same data as Figure 4 in the main text. The “+” symbols indicate the medians. Similar trends are observed for all three considered edge weights.

**
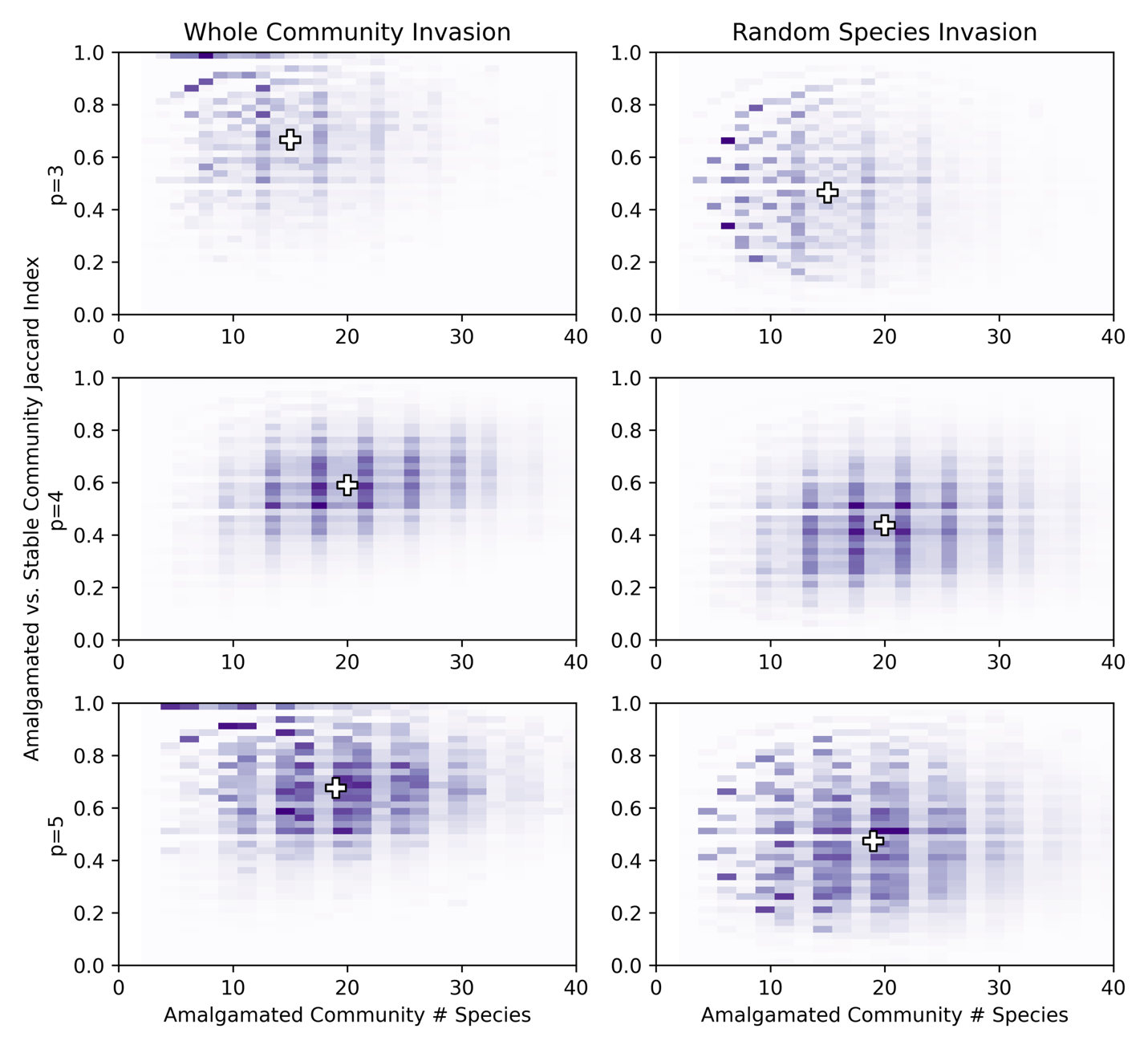
**

**Figure C.** The Jaccard index vs. the total number of native and invasive species at the time of invasion. The middle row panels show the same data as Figure 5 in the main text. The “+” symbols indicate the medians. For all three considered positive edge weights, whole community invasion results in less species turnover than in the case of random species invasion. The lack of high or low Jaccard indices in the case of small amalgamated communities and random species invasions indicates that some random invaders are able to establish, but that the original community structure is not completely destabilized.

**
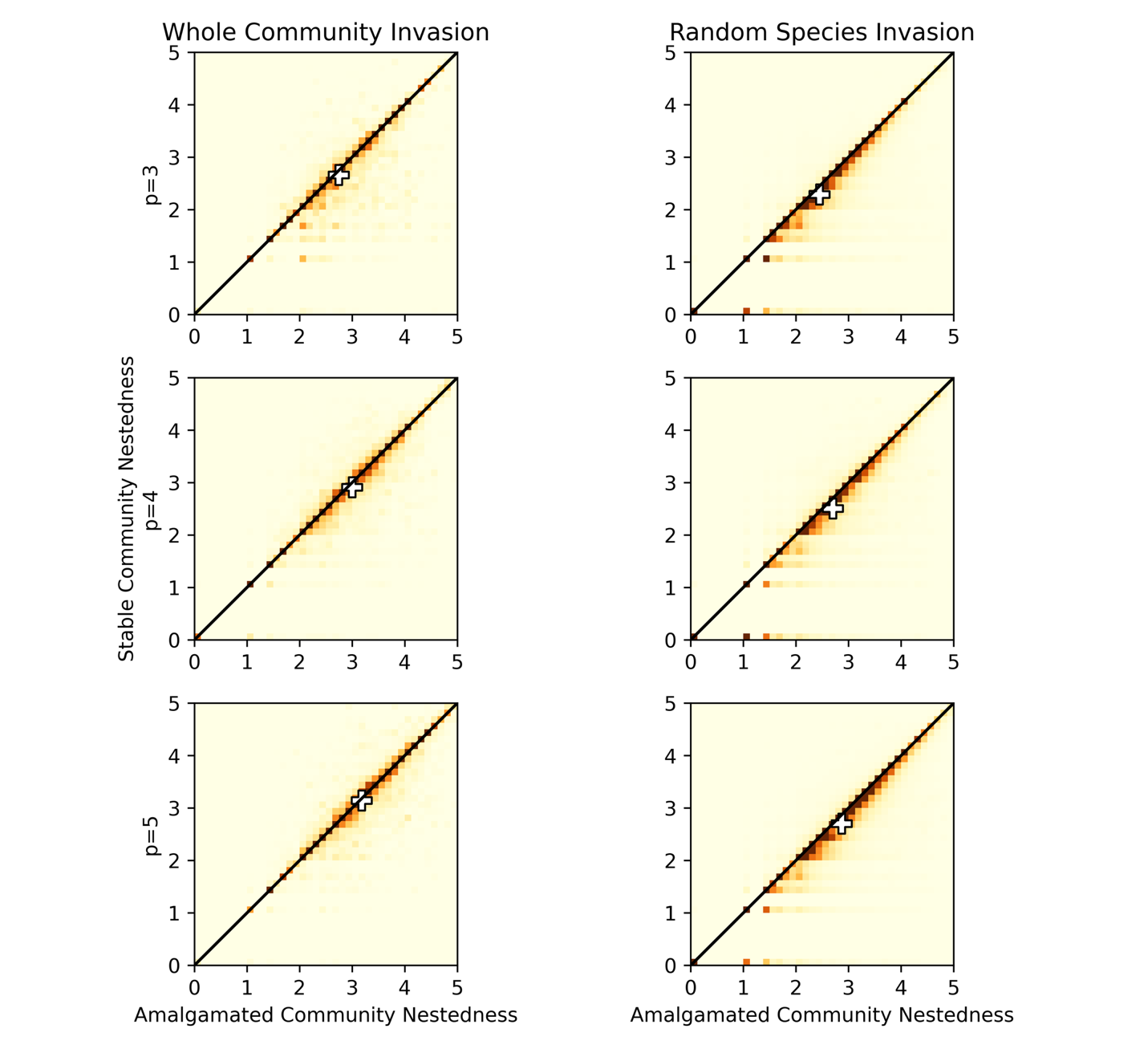
**

**Figure D.** The nestedness of amalgamated vs. final stable communities. The middle row panels show the same data as the top row of Figure 6 in the main text. The “+” symbols indicate the medians. In all cases the nestedness decreases slightly as a result of the invasion.

**
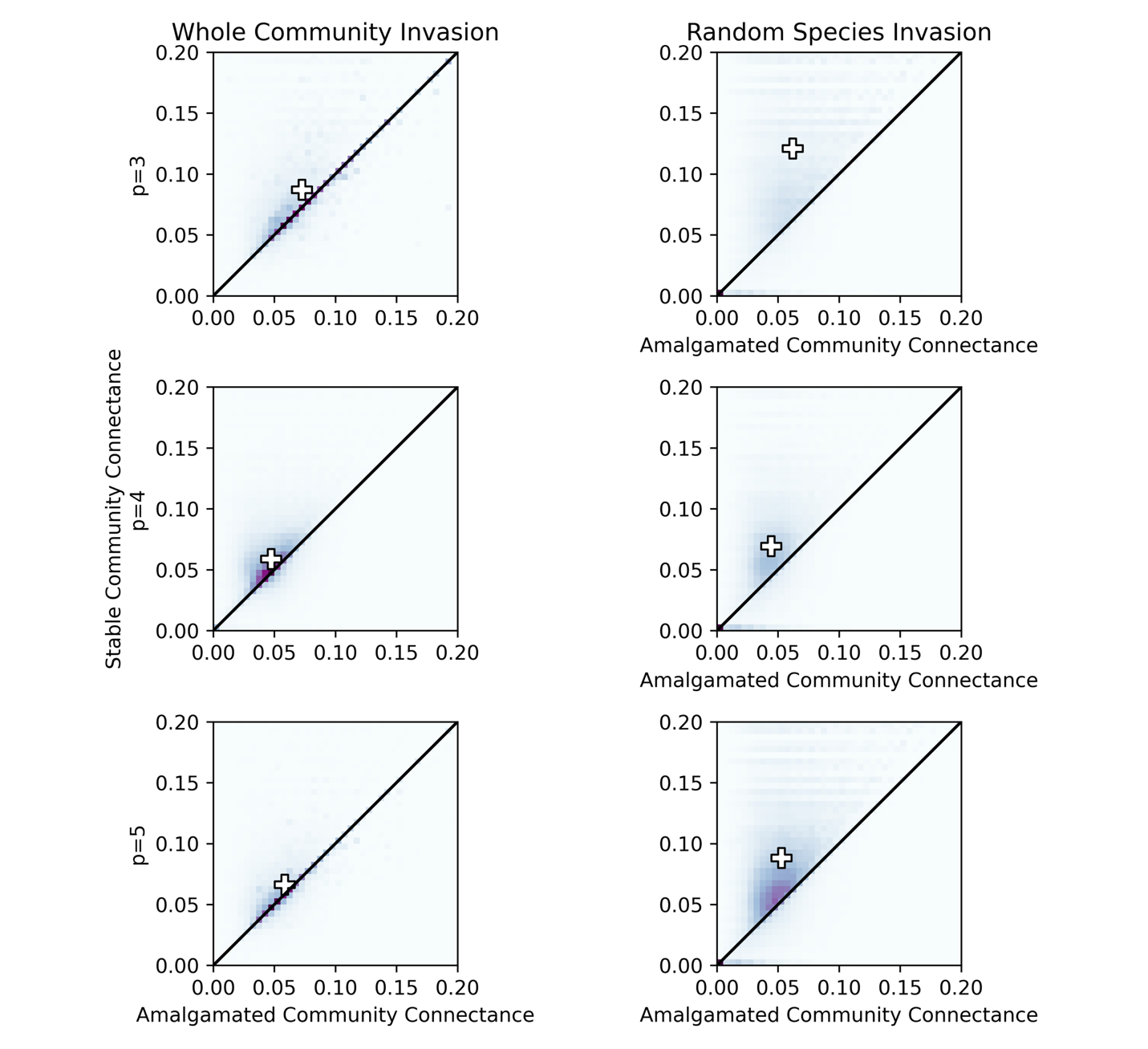
**

**Figure E.** The connectance of amalgamated vs. final stable communities. The middle row panels show the same data as the bottom row of Figure 6 in the main text. The “+” symbols indicate the medians. In all cases the connectance increases as a result of the invasion.

**
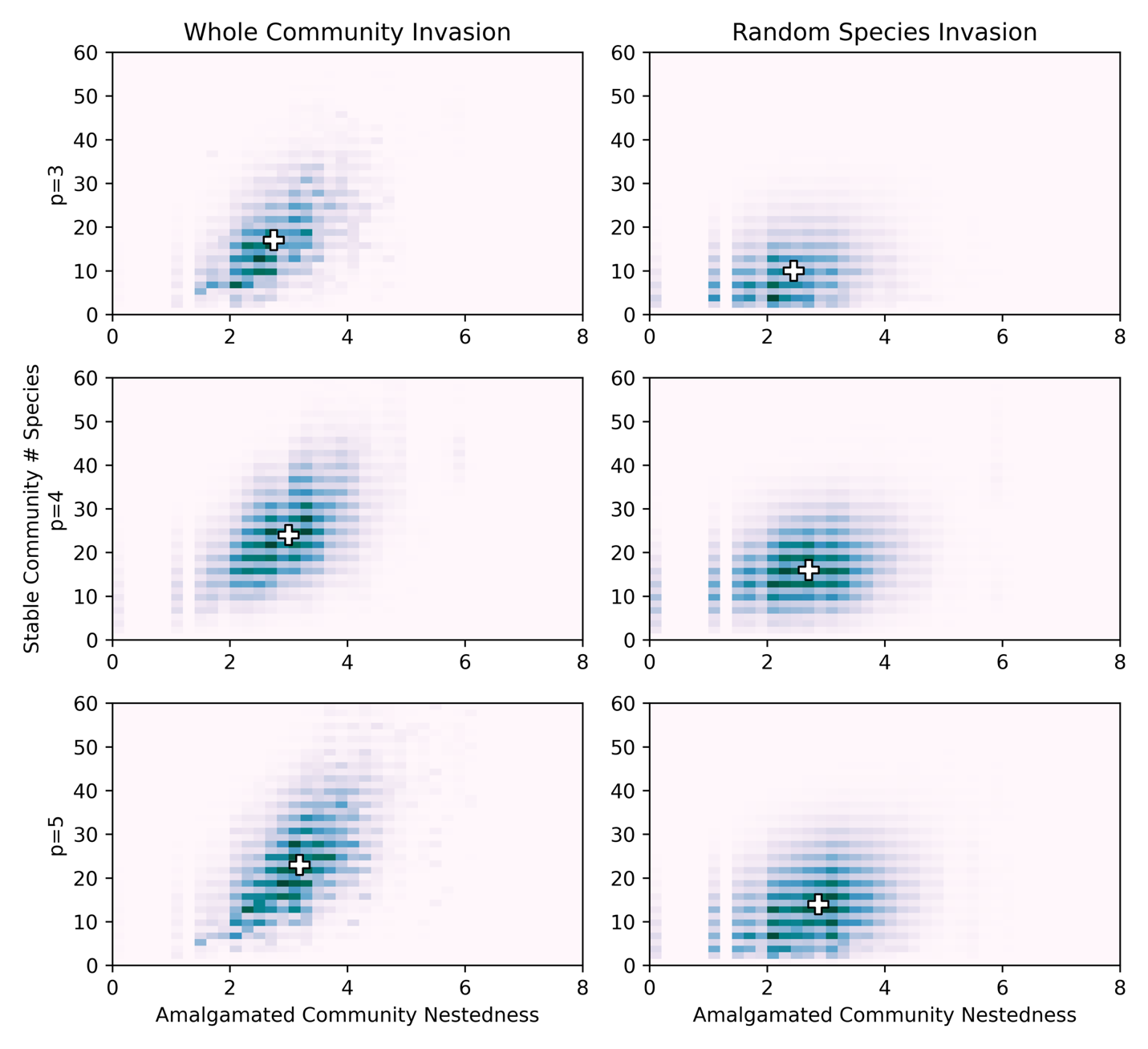
**

**Figure F.** The nestedness of amalgamated communities vs. final stable community size. The middle row panels show the same data as the top row of Figure 7 in the main text. The “+” symbols indicate the medians. The behavior is qualitatively consistent for all three considered edge weights.

**
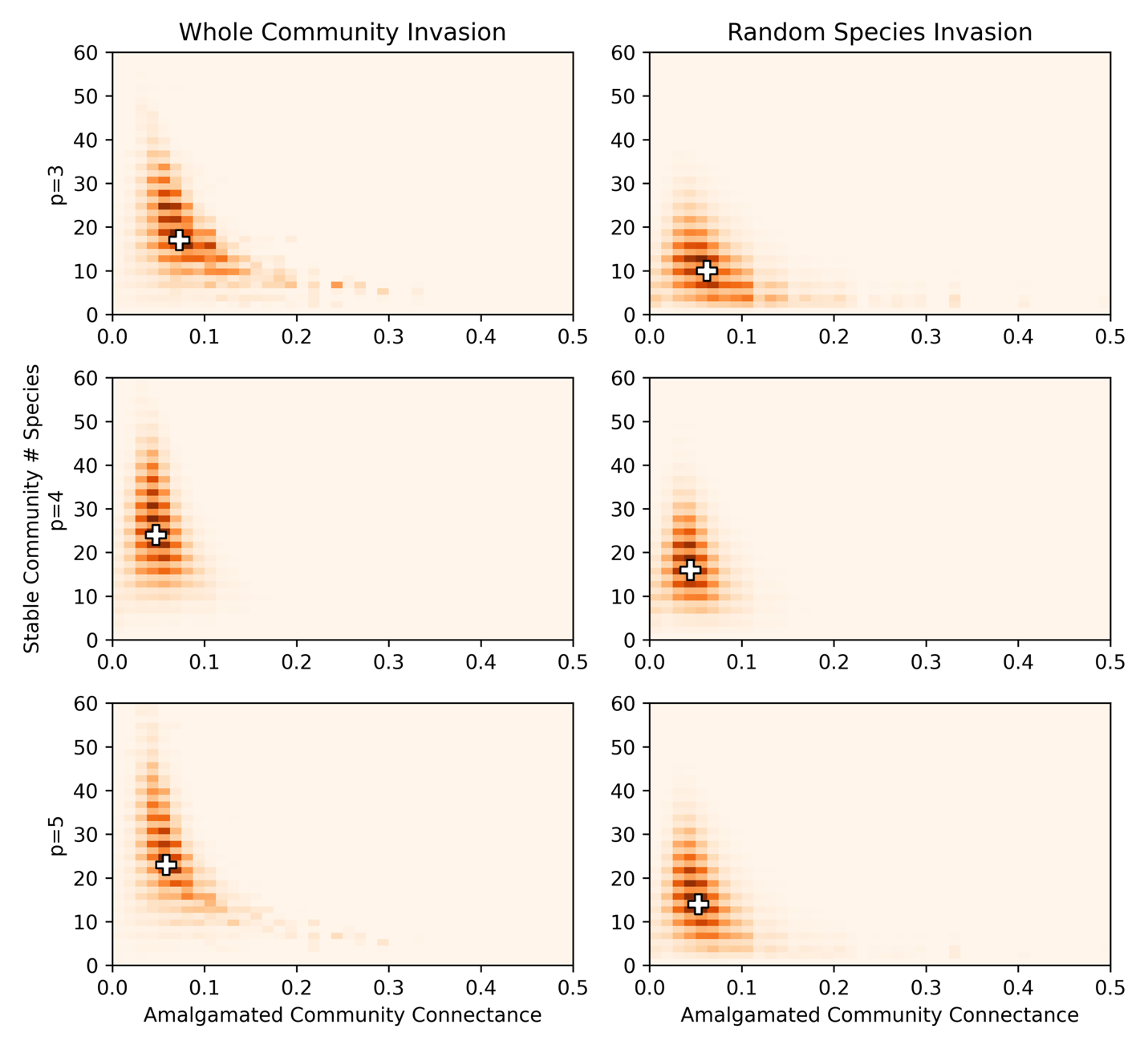
**

**Figure G.** The connectance of amalgamated communities vs. final stable community size. The middle row panels show the same data as the bottom row of Figure 7 in the main text. The “+” symbols indicate the medians. The negative correlation reported in the main text for *p*=4 is also observed for *p*=3 and *p*=5.
